# Ultrasensitive HCV RNA Quantification in Antiviral Triple Therapy: New Insight on Viral Clearance Dynamics and Treatment Outcome Predictors

**DOI:** 10.1371/journal.pone.0158989

**Published:** 2016-08-25

**Authors:** Anna Rosa Garbuglia, Ubaldo Visco-Comandini, Raffaella Lionetti, Daniele Lapa, Filippo Castiglione, Gianpiero D’Offizi, Chiara Taibi, Marzia Montalbano, Maria Rosaria Capobianchi, Paola Paci

**Affiliations:** 1 Laboratory of Virology, “Lazzaro Spallanzani” National Institute for Infectious Diseases, IRCCS, Rome, Italy; 2 Clinical Department, “Lazzaro Spallanzani” National Institute for Infectious Diseases, IRCCS, Rome, Italy; 3 Istituto per le Applicazioni del Calcolo (IAC)—CNR, Rome, Italy; 4 Istituto di Analisi dei Sistemi ed Informatica “Antonio Ruberti” (IASI)—CNR, Rome, Italy; Chiba University, Graduate School of Medicine, JAPAN

## Abstract

**Objectives:**

Identifying the predictive factors of Sustained Virological Response (SVR) represents an important challenge in new interferon-based DAA therapies. Here, we analyzed the kinetics of antiviral response associated with a triple drug regimen, and the association between negative residual viral load at different time points during treatment.

**Methods:**

Twenty-three HCV genotype 1 (GT 1a n = 11; GT1b n = 12) infected patients were included in the study. Linear Discriminant Analysis (LDA) was used to establish possible association between HCV RNA values at days 1 and 4 from start of therapy and SVR. Principal component analysis (PCA) was applied to analyze the correlation between HCV RNA slope and SVR. A ultrasensitive (US) method was established to measure the residual HCV viral load in those samples which resulted “detected <12IU/ml” or undetectable with ABBOTT standard assay, and was retrospectively used on samples collected at different time points to establish its predictive power for SVR.

**Results:**

According to LDA, there was no association between SVR and viral kinetics neither at time points earlier than 1 week (days 1 and 4) after therapy initiation nor later. The slopes were not relevant for classifying patients as SVR or no-SVR. No significant differences were observed in the median HCV RNA values at T0 among SVR and no-SVR patients. HCV RNA values with US protocol (LOD 1.2 IU/ml) after 1 month of therapy were considered; the area under the ROC curve was 0.70. Overall, PPV and NPV of undetectable HCV RNA with the US method for SVR was 100% and 46.7%, respectively; sensitivity and specificity were 38.4% and 100% respectively.

**Conclusion:**

HCV RNA “not detected” by the US method after 1 month of treatment is predictive of SVR in first generation Protease inhibitor (PI)-based triple therapy. The US method could have clinical utility for advanced monitoring of virological response in new interferon based DAA combination regimens.

## Introduction

In 2011, the first direct-acting antiviral drugs (DAA), telaprevir (TPV) and boceprevir (BCV) were approved for treatment of chronic HCV genotype 1 infection [1–5]. HCV RNA measurements at baseline and during treatment were used to define the optimum moment for early discontinuation of treatment in virological non-responders and the treatment duration for patients responding to therapy (response guided therapy, RGT). Treatment with TPV, for example, should be discontinued at T12 week (T12w), if HCV RNA is >1,000 IU/ml at T12w [6,7]. Several studies underlined the importance of accurate quantification of the HCV RNA viral load when making decisions about shortening or extending treatment [8–9]

The stopping rules are based on viral load data obtained during the clinical phase II/III studies using the High Pure System/COBAS TaqMan v2 (Roche, Molecular Diagnostics, Pleasanton, Ca, USA). In fact, in the definition of sustained virological response (SVR) for patients treated with a triple combination of TPV/PEG-IFN/RBV, a viral load <25IU/ml at T24 w after EOT was chosen to establish the success of the therapy [10, 11]. Recently, the importance of HCV RNA decline in early phases of treatment as a predictor of SVR has also been observed in triple therapy with simeprevir, Peg-interferon and ribavirin in patients infected with genotype 1 [12]

Nevertheless, during recent years, several HCV RNA detection and quantification assays have been licensed Artus HCV_QS-RGQ (Qiagen GmbH, Hilden, Germany), Roche COBAS Ampliprep/COBAS TaqMan v1 and v2, Roche High Pure System/COBAS TaqMan HPS (Roche Molecular Diagnostics, Pleasanton, Ca, USA), ABBOTT RealTime HCV (Abbott Molecular Inc, Des Plaines, IL, USA), Siemens Versant HCV, v1.0 (Siemens Healthcare Diagnostics Inc., Tarrytown, NY, USA) with different limit of detection (LOD), and lower limit of quantification (LLOQ). Several independent head-to-head comparison studies of quantitative HCV RNA assays, showed differences in the analytical performance with respect to precision, accuracy, and sensitivity at the low end of the measurement range [13–16]. A retrospective analysis of SVR based on virological response at T4w of triple therapy showed lower SVR rates in patients with a shortened treatment duration (24/28 weeks), with residual viraemia (detectable HCV RNA, but <25 IU/ml) compared to those with completely undetectable HCV RNA [17]. Conversely, another study [14] described that the rapid virological response (RVR) rate was significantly different when assessed by Cobas Ampliprep/CobasTaqMan and Abbott RealTi*me* HCV with substantial impact on an abbreviated course of the therapy (24 weeks). In this retrospective study, after exploring the correlation of HCV RNA at baseline, HCV RNA decline and RVR and EVR response, we evaluated the predictive value of HCV RNA <12 IU/ml on SVR, quantified with an ultrasensitive (the US method), in HCV genotype 1 infected patients treated with Telaprevir/Peg-interferon/ Ribavirin (TPV/PEG-IFN/RBV).

## Materials and Methods

### Study design

HCV genotype 1 infected chronic patients who were either HCV treatment naive or who had previously experienced treatment failure to PEG-IFN/RBV (standard of care, SOC) therapy were included in this retrospective study. The criteria used to determine hepatitis C therapy followed the EASL Recommendations on treatment of Hepatitis C from 2014 [10].

The baseline data collected on each patient included demographic data (gender, age, BMI), HCV RNA viral load, and fibrosis stage.

All patients received a combination treatment of TPV/ PEG-IFN/RBV for 12 weeks, followed by an additional 12 weeks of PEG-IFN and RBV in naive subjects who achieved an extended rapid viral response (eRVR), and 36 weeks of PEG-IFNα2b and RBV in the other cases. SVR was defined as serum HCV RNA not detected at 24 weeks post treatment. For further details on efficacy of treatment see S1 Data.

The study was designed and performed according to the Helsinki declaration, and we received ethical approval from” Institutional Review Board of INMI L Spallanzani, IRCCS”, Rome (statement n.32/06-16-2011). All patients provided written informed consent before participating in this study.

### HCV RNA detection

HCV RNA plasma quantification or concentration was performed on plasma samples drawn at day 0 (pre-dose-baseline), 1, 2, 3, 4, 5, 14, 28, and at weeks 8, 12, 24 of treatment. In addition, plasma samples were drawn during the follow-up period, at weeks 4, 8, 12, 16, 20, 24, after end of therapy (EOT). Day 1 was defined as 1 day after the date of starting TPV treatment.

Plasma HCV RNA values were quantified using the ABBOTT RealTime HCV assay (Abbott Molecular Inc, Des Plaines, Il, USA, lower limit quantification (LLOQ) = 12 IU/ml, and LOD 8.3IU/ml for genotype 1) [18]. This assay can detect HCV RNA concentrations lower than 12IU/ml, with a detection probability of <95%. Values below the LLOQ were reported as detected <12 IU/ml.

A quantitative result below the LLOQ was also determined by a ultrasensitive (US) residual viral load assay (see below).

### IL28B rs12979860 polymorphism genotyping

Single nucleotide polymorphism (SNP) rs 129679860 (on chromosome 19q13), located 3 kilobases upstream of the IL28B gene was genotyped. Genotyping was carried out using a custom TaqMan assay (Applied Biosystem, Foster City, California, USA) on DNA isolated from blood or serum. The assay conditions are described in Visco et al [19].

The researcher responsible for genotyping blinded to other patient data. The IL28B genotyping were defined as C/C or non C/C (T/T or C/T). IL28B C/C genotype has been strongly associated with SVR in patients with HCV genotype 1 [20, 21].

### HCV RNA ultrasensitive protocol

The following modifications were introduced in the standard procedure of the Abbott RealTime HCV 1 assay: 1) an extended calibration curve towards lower HCV RNA concentrations; 2) the use of higher patient sample volumes that were concentrated via ultracentrifugation; 3) the use of a reduced volume of internal control, 4) the use of an adopted “open-mode” software for quantification (S2 Data, S2 Fig). For method validation, MIQE guidelines were considered [22].

### Statistical analysis

Linear regression analysis was employed to analyze HCV RNA kinetic considering the ultrasensitive results.

### Slope definition

The slope *s*(*t_i_*) of the viremia measurements at the time step *t_i_* is defined as

$$s(t_{i})=\frac{y(t_{i+1}-t_{i})-y(t_{i})}{t_{i+1}-t_{i}}\;i=0,\ldots ,4$$

with

$$t_{i}=\{t_{0},t_{1},t_{2},t_{3},t_{4},t_{5}\}=[T0,T1,T4,T1m,T2m,T3m]$$

where T0, T1, T4 correspond to day 0, 1, 4 days of treatment; T1m, T2m, T3m correspond to 1, 2, 3 months of treatment, respectively; and

$$y(t_{i})=\log(x(t_{i}))\;i=0,\ldots ,5$$

where *x* (*t*_*i*_) is the viremia measured as HCV RNA at time point *t*_*i*_.

### ANOVA test to identify inter- and intra-classes slope differences

In order to evaluate whether statistically significant differences intra- and inter-classes existed between the slopes of HCV RNA ultrasensitive measurements, the ANOVA test (Analysis Of Variance) was performed.

### Automatic data classification

There are many possible techniques that can be used for data classification. In this study, in order to classify the patients’ data as EVR /EVR/SVR on the basis of the available viremia, we used the Linear Discriminant Analysis (LDA) [23] and Principal Component Analysis (PCA) [24–26]. Important differences exist between these two methods.

First, the objective of LDA is to maximize class discrimination, whereas the objective of PCA is to squeeze variance into as few components as possible. Second, LDA produces exactly as many linear functions as there are classes, whereas PCA produces as many linear functions as there are original variables. Third, principal components are always orthogonal to each other ("uncorrelated"), while that is not generally true for LDA linear scores. Note that LDA maximizes the ratio of between-class variance to within-class variance in any particular data set, thereby guaranteeing maximal separability.

The routine LDA from MATLAB was used to classify patients on the basis of the viral HCV RNA at T1 (day one) and T4 (day four).

PCA is a statistical procedure that uses an orthogonal transformation to convert a set of observations (correlated variables) into a set of linear independent variables called principal components. The number of principal components is less than or equal to the number of original variables. This transformation is defined in such a way that the first principal component has the largest possible variance and each succeeding component in turn has the highest variance possible under the constraint that it is orthogonal to (i.e. uncorrelated with) the preceding components.

Throughout this analysis we aimed to determine if there was a correlation between the magnitude of the slope and EVR/RVR/SVR response.

### Association between reduced serum HCV RNA levels at different time points of treatment and SVR

Receiver-operating characteristics (ROC) analyses were performed to determine cut-off values for sensitivity and specificity for predicting SVR. Furthermore, negative predictive power (NPV), positive predictive power (PPV), specificity and or predicting SVR were calculated [27]. Statistical analysis was performed using a MATLAB routine named *ROCout*. The analysis was carried out considering a) the HCV RNA values obtained by ABBOTT standard procedure (ART), and b) also considering the available results obtained with the US method data, to evaluate the association between reduced serum HCV RNA levels at months 1, 2, and 3 after starting therapy and SVR.

## Results

### Baseline characteristics

Twenty-three HCV genotype 1 (GT 1a n = 11; GT1b n = 12) infected patients were included in this retrospective study. Seventeen patients had been previously treated with standard therapy. Baseline patient characteristics are shown in Table 1. All patients had advanced liver fibrosis (Metavir F3-4) and 47.8% (11/23) showed hepatic cirrhosis. No differences in demographic, biochemical, or hematological feature, and severity of liver fibrosis (F3 or F4) were observed.

**Table 1:** Baseline characteristics of patients.

Gender, male (%)	62%
Age (years), mean ± SD	59.0 ± 7.0
BMI (Kg/m^2^), mean ± SD	24.8 ± 3.39
GOT (mU/ml),mean ± SD	87.6 ± 54.5
GPT (mU/ml),mean ± SD	115 ± 69.6
Albumin g/dl,mean ± SD	4.40 ± 0.4
Platelets x10^3^/mmc, mean ± SD	150.7 ± 48.5
Hb g/dl, mean ± SD	15.0 ± 0.9
T0 VL HCV (IU/mL)	1,224627± 1,449927.16
Fibrosis F 3* (%)	52%
Fibrosis F 4* (%)	48%
Hepatic Cirrhosis (%)	52%
Genotype: C/C; C/T; T/T	2; 13; 8
**Pre-Telaprevir therapy:** Naïve; Partial responder; Non responder; BreakThrough; Relapser	4; 2; 9; 1; 7

*Liver fibrosis stage was determined by liver transient elastography (Fibroscan, Echosen, Paris), it was established according to METAVIR score (the French METAVIR cooperative Study Group, 1994), and was stratified as mild (F1-F2) and advanced (F3-F4).

### Virologic response

Sixteen out of 23 patients completed the phase of telaprevir-based triple therapy and all but one were undergoing dual therapy with PEG-IFN and RBV at the end of observational period. The remaining 7 patients discontinued the therapeutic regimen due to side effects, mainly because of severe anemia, skin rash or bacterial infections.

TPV caused a rapid decrease in HCV RNA levels after the initiation of treatment in all patients. The average changes in HCV RNA at day 1 were -2.43 log IU/ml in patients with RVR and -2.54 log IU/ml in patients with EVR; at day 5, -3.39 log IU/ml and -3.27 log IU/ml in RVR and EVR patients, respectively. No statistically significant diversity was observed between the two groups (t-test p-value > 0.05). We observed RVR in 14 patients with HCV RNA <12 IU/ml (calculated with ART), and 9 EVR; 2 patients were always HCV RNA detected. Overall, there were 14 SVR patients. No significant differences were observed at T0 in HCV RNA median values among the patients who achieved SVR and no-SVR patients (S1 Fig), p-values of a two-sided Wilcoxon rank sum test >0.05). Among the patients who stopped TPV treatment prematurely, 3/5 showed SVR response. According to prior response, the SVR rates were: 75% (3/4 patients) in treatment-naive patients, 85% (6/7 patients) in previous relapsers, 0% (0/2 patients) in partial responders, and 50% (5/10) in null-responders/breakthrough patients.

None of the patients experienced a viral relapse between SVR12w and SVR24w time points.

### IL28B rs12979860 polymorphism

Two out of 23 patients showed IL28B rs 12979860 C/C genotype (Pt12, non-responder in previous treatment; Pt20-naive), and both achieved SVR. Thirteen patients had C/T genotype and 8 T/T alleles (Table 1). Among those with a non C/C genotype, the SVR was 53.8% (7/13) in patients with the IL28B C/T genotype and 50% (4/8 patients) with an IL28B T/T genotype (Table 1).

### The HCV RNA US method results

Forty-seven samples, that resulted HCV RNA detected <12 IU/ml or HCV RNA that was undetected with the ART method, were retested with the US method. Accordingly to US method values we were able to recalculate the average of patients with values detected <12 IU/ml or “not-detected” values at T4w, T8w, and T12w.

At T4w, among the 12 samples analyzed, 7 patients (7/12, 58.3%) considered as RVR with ART were positive with the US method; HCV RNA was “not detected” with both methods in five patients (5/12, 41.7%), detected with the ultrasensitive method and detected below the lower limit of quantification (BLLOQ) in 6 patients (6/12, 50%); only 1 patient was detected with the US method and “not detected” with ART.

At T8w, 15 patients were analyzed with both methods and we observed 86% of concordance. Nine samples were HCV RNA “not detected” with both methods (9/15, 60%), 4 patients showed a HCV RNA “detected”, quantifiable at 6.8 IU/ml (mean value) with the US method and detected <12 IU/ml with ART. One sample was detected (positive <4 IU/ml) with the US method and undetected with ART, instead another sample was detected < LLOQ (detected <12) with ART and not detected with the US method. At T12w concordance was 100%; and the overall concordance in either not detecting or detecting any HCV RNA was 89.8% (Table 2).

**Table 2:** HCV quantification with Abbott RealTi*me* standard method (ART) and ultrasensitive method (US) in all analyzed samples.

ART Method		Abbott US		
		No detected	Detected (<4 IU/ml)	Detected (≥4 IU/ml)
No detected	n = 31	n = 27	n = 4	n = 0
Detected (<12 IU/ml)	n = 16	n = 1	n = 6	n = 9
Detected (<12 IU/ml)	*n = 2	n = 0	n = 0	n = 2

n is the number of samples. Only two samples with HCV RNA value >12 IU/ml by ART method were retested with US method to check the reproducibility of HCV RNA quantification upper 12 IU/ml by the US method. The obtained results were comparable.

Among the patients classified as RVR by ART, 35.7% (5/14) of them achieved HCV RNA “not detected” by the US method at T4w, and all 5 five patients achieved SVR. One out of 5 patients was naïve, 3 were relapsers and 1 was a non-responder in SOC therapy. One relapser patient suspended Peg-IFN/RBV administration at T24w for eresipela; nevertheless, achieving SVR status.

### Viral kinetics

**Fig 1:**
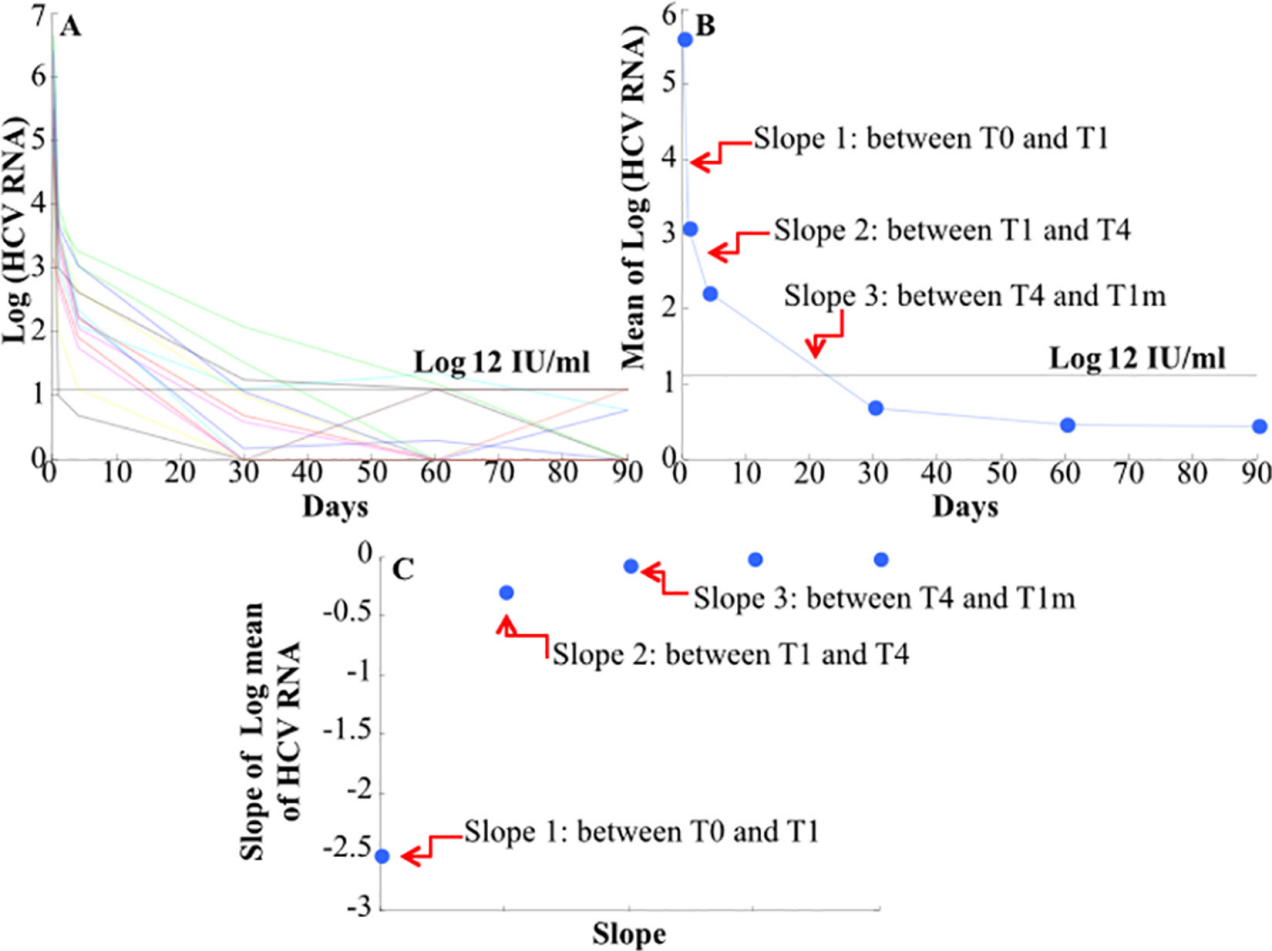
The temporal trend of HCV RNA for 14 patients with available daily serum samples; values are expressed in Log IU/ml. We also reported values <12 IU/ml determined by ultrasensitive assay. **B**: Average of Log (HCV RNA) measurements; the red arrows indicate that three different slopes appear within the first month (T1m). Blue dashed lines correspond to the undetectable limits with the standard measurement of HCV RNA. The first slope falls within the first day (T1) and the second slope falls within the fourth day (T4). **C**: mean slopes of HCV RNA measurements observed during the first three months of treatment. T0, T1, and T4 correspond to day 0, 1, 4 days of treatment; T1m, T2m, T3m correspond to months 1, 2, 3 of treatment, respectively.

We analyzed data from 14 patients with an available daily HCV RNA serum sample. Data consisted of HCV RNA at the time of enrolment (T0), after one day (T1), after four days (T4), after one month (T1m), after two months (T2m) and after three months (T3m) of initiating therapy (Fig 1A). Three slopes are evident within the first month. The first slope falls within the first day, the second slope falls within the fourth day and the third slope falls within the first month (Fig 1B).

Then, we evaluated the slope of the HCV RNA by using the ultrasensitive data and we found 5 slopes within the first three months (Fig 1C).

### ANOVA test

**Fig 2:**
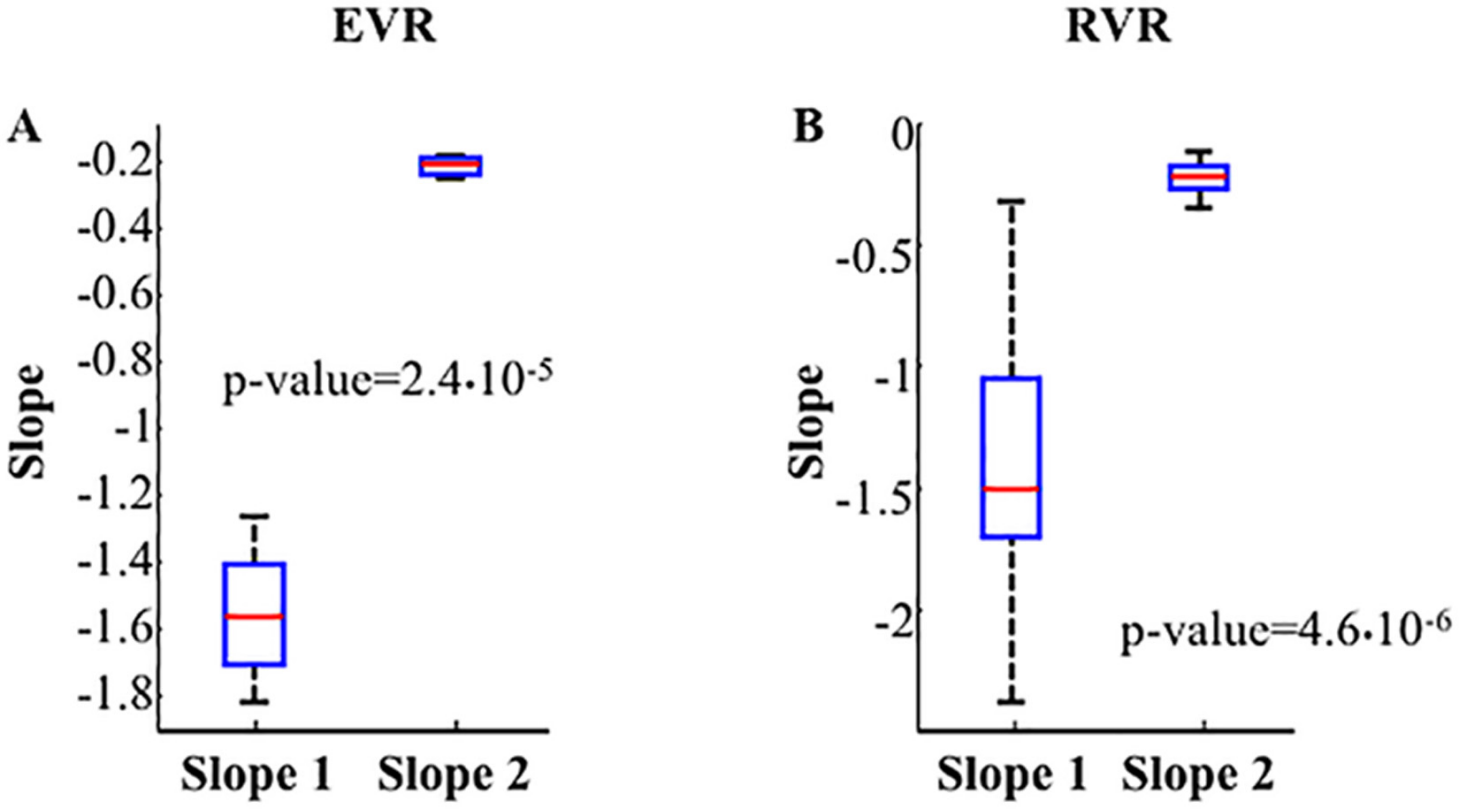
Box plot for EVR (**Panel A**) and RVR (**Panel B**) of the first two slopes of HCV RNA measurements for the 14 patients. The top and bottom of the blue boxes are the first (25th percentile) and the third (75th percentile) quartiles; the red band inside the boxes is the second quartile (i.e., the median); the dashed lines extending vertically from the boxes (called whiskers) indicate data outside the interquartile range that are not outliers. Points are drawn as outliers if they are larger than q3 + w(q3 –q1) or smaller than q1 –w(q3 –q1), where q1 and q3 are the 25th and 75th percentiles, respectively. We used w = 1.5 that corresponds to approximately +/–2.7σ and 99.3 coverage if the data are normally distributed. The plotted whisker extends to the extreme values, which are the most extreme data values that are not outliers. The resulting p-values of a two-sided Wilcoxon rank sum test performed between the first two slopes within each group of patients are shown in black.

Classifying patient as EVR and RVR, we found that the inter-group slopes were not significantly different (p-value >0.05). On the contrary, the two-sided Wilcoxon rank sum test resulted significant for the first two slopes within each group (p-value<0.05) both for the EVR group (Fig 2A) and the RVR group (Fig 2B).

### Principal Component Analysis (PCA)

**Fig 3:**
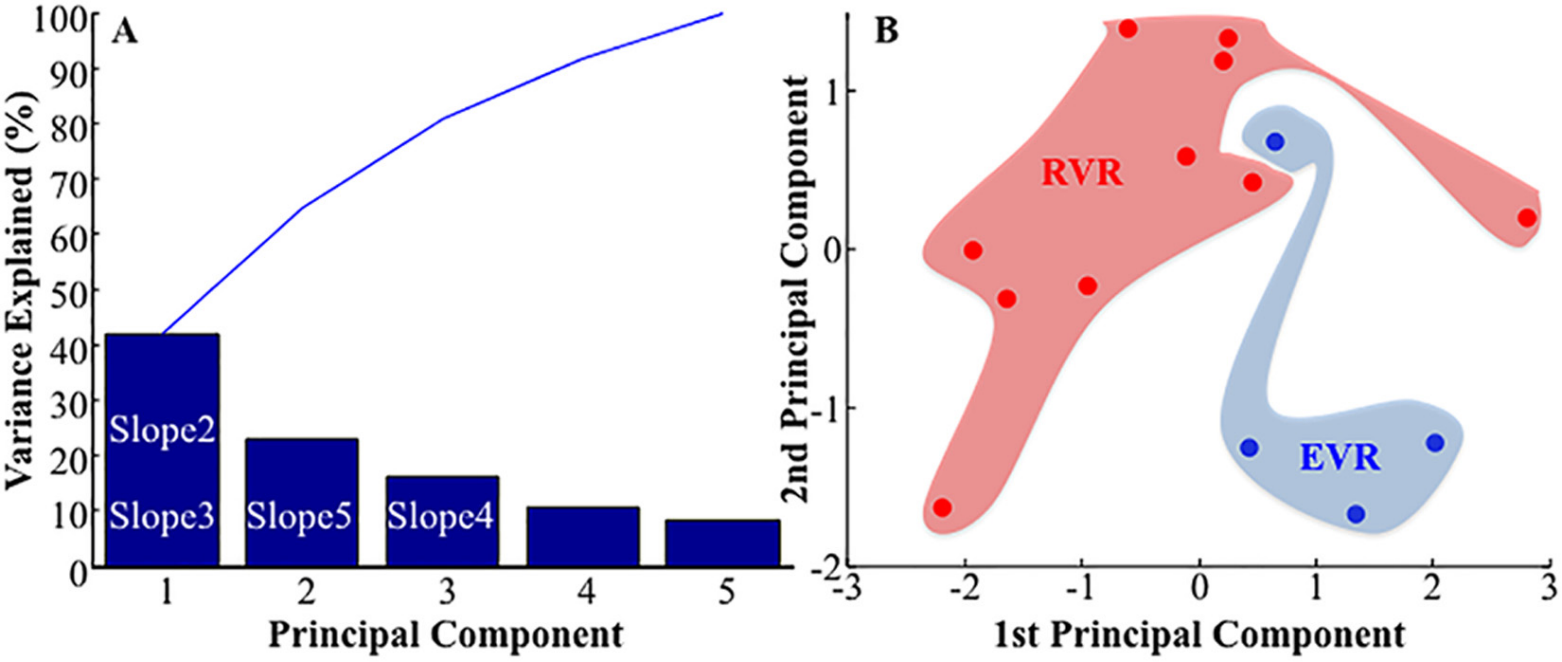
**Panel A**: This plot displays the percent variability explained by each principal component and is called *Pareto chart*. It is a type of chart that contains both bars and a line graph, where individual values are represented in descending order by bars, and the line represents the cumulative total. In particular, the y-axis represents the percentage of the data variance explained by each principal component, whereas the x-axis represents the principal components that are able to explain the first 100% of the cumulative distribution. The principal component analysis is performed using the slopes of the HCV RNA values for the 14 patients. The first principal component is able to explain more than the 40% of the variance explained and the first three components are able to take into account more than 80% of the cumulative distribution. Looking at the PCAs’ loadings we found that the first principal component shows high correlation with the second and the third slope, while the second principal component correlates with the fifth slope and the third component correlates with the fourth slope. This indicates that the first slope, i.e., one day after the enrolment, does not contribute to the total variance. Inside each bar, the white writings represent which slopes correlate with each principal component. **Panel B**: This plot represents a scatter plot (*score plot*) of the projection of the data (i.e. the HCV RNA values for the 14 patients) onto the first two PCs; the x-axis contains the first PC while the y-axis contains the second PC. In this plot it is possible to group patients as EVR or RVR. This means that the first two principal components are able to classify patients as EVR or RVR. Since the first principal component correlates with the second and third slope (see panel A) and the second principal component correlates with the fifth slope (see panel A), we can conclude that these three slopes are necessary to classify patients as EVR or RVR.

We performed PCA on the slopes *s*(*t*_*i*_), *i* = 0,…,4, of the HCV RNA for the 14 patients with daily plasma sample collection within the first week of therapy (Fig 3A). The first principal component explains about 40% of the variance whereas the first three principal components together explain roughly two-thirds of the total variability in the standardized ratings.

Looking at the PCAs’ loadings we found that the first principal component shows high correlation with the second and the third slope (*s*(*t*_*1*_) and *s*(*t*_*2*_) respectively), while the second principal component correlates with the fifth slope *s*(*t*_*4*_) and the third component correlates with the fourth slope *s*(*t*_*3*_) (data no shown). This indicates that the first slope, *s*(*t*_*0*_), i.e., one day after the enrolment, does not contribute to the total variance.

**Fig 4:**
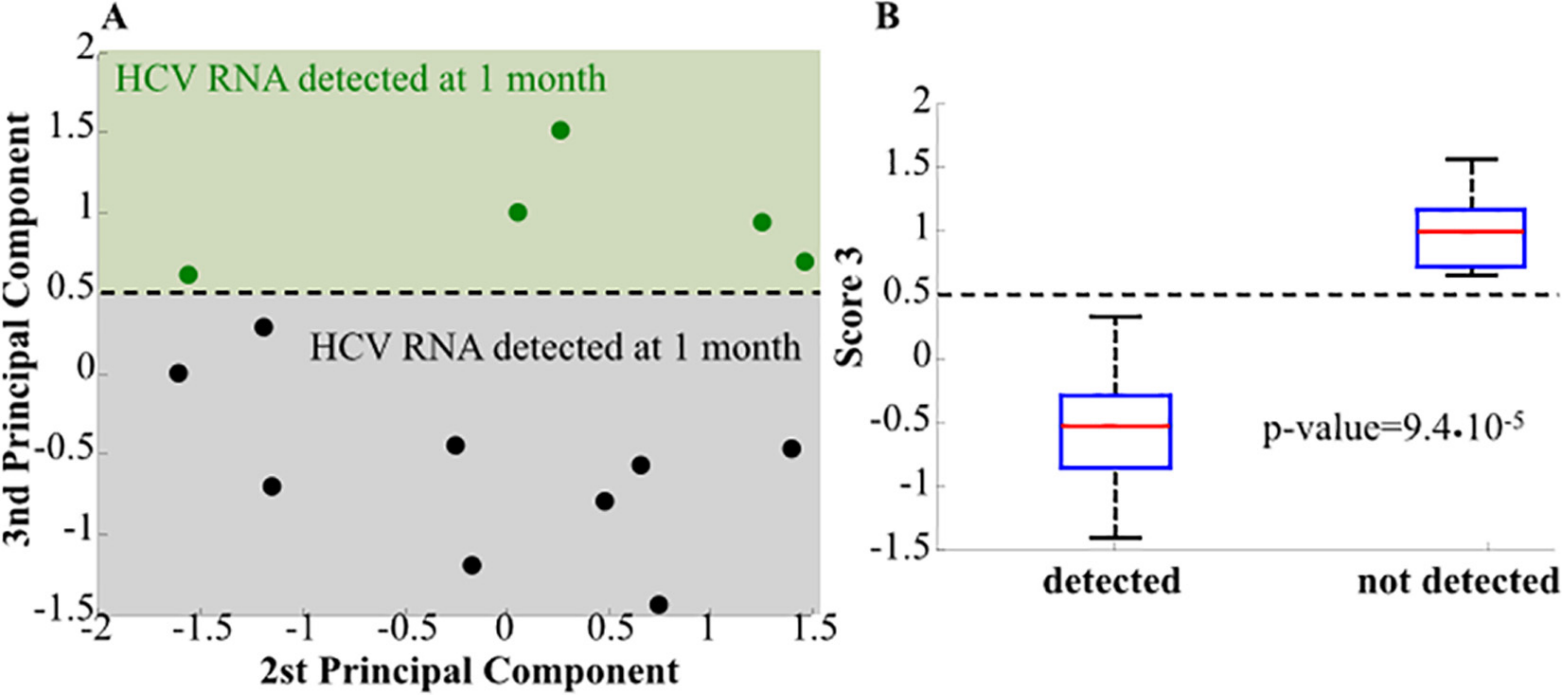
**Panel A**: Score plot where the x-axis contains the second PC while the y-axis contains third PC. **Panel B**: Box plot of the third score–that is the projection of the data on the third PC- for *detected* and *undetected* patients at month 1 after therapy initiation as results from the ultrasensitive assay. The resulting p-values of a two-sided Wilcoxon rank sum test are shown. This plot and the p-value show that the second and third PC shown in panel A, are able to separate *detected* and *undetected* patients at month 1 after therapy initiation in a statistically significant way.

Looking at the score plot in Fig 3B, i.e., the projection of the original data onto the principal components in two dimensions, we can observe that the first two principal components are able to separate the two classes of patients, EVR and RVR. Since the first principal component correlates with the second and third slope and the second principal component correlates with the fifth slope, we can conclude that these three slopes are necessary to classify patients as EVR or RVR. On the contrary, the score plot in Fig 4A shows that second and third principal components are able to well separate patients in HCV RNA “detected” and “not detected” at T1m (*i*.*e*., classification on the basis of an US at T1m after therapy initiation). This is not surprisingly since the second and the third principal components correlate with the fifth and fourth slopes, respectively. This correlation is statistically significant (p-value<10^−4^) as shown in Fig 4B. Furthermore, none of these slopes correlated with SVR (data not shown).

### Linear Discriminant Analysis (LDA)

**Fig 5:**
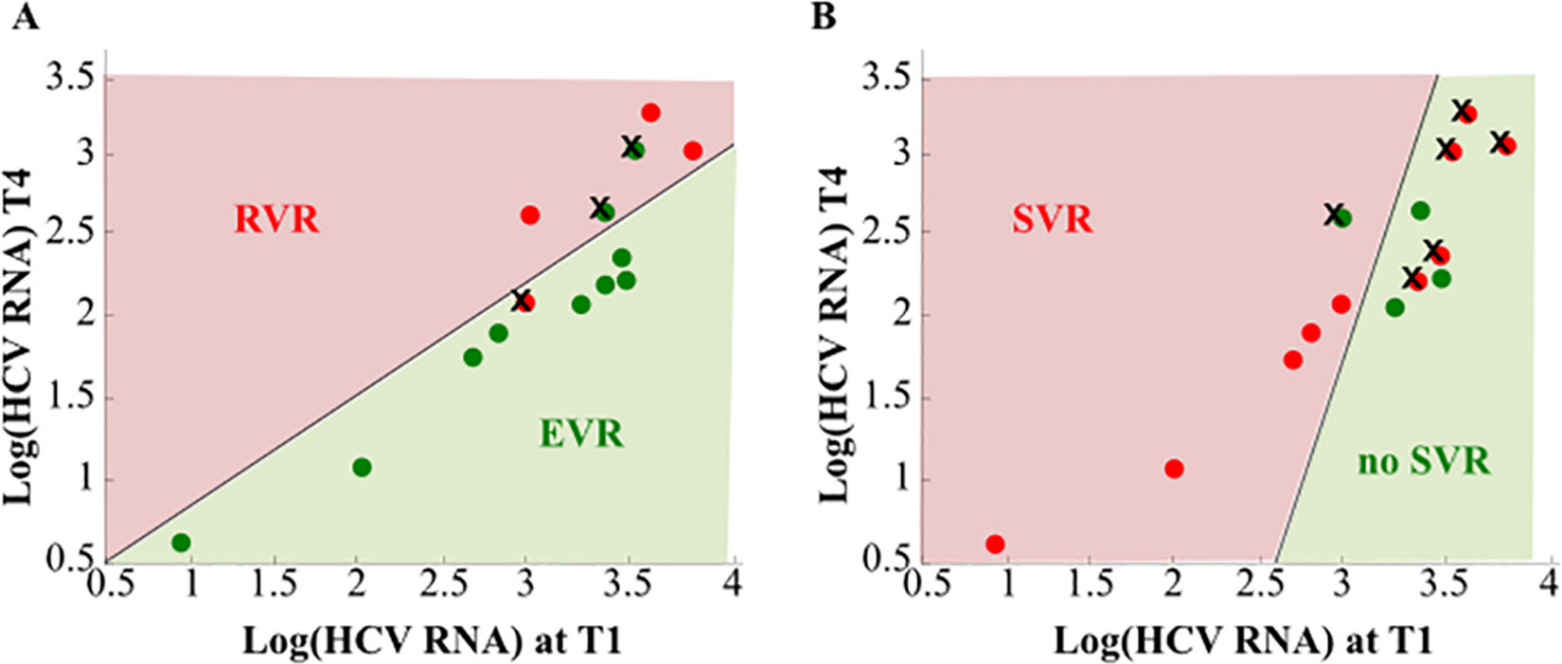
Linear discriminant analysis of viral Log (HCV RNA) for the classes EVR and RVR (**Panel A**) and class SVR (**Panel B**). The black line represents the decision region separating data; red region corresponds to EVR (or SVR) patients whereas green region corresponds to the RVR (or no-SVR) patients. Red circles and green squares represent Log (HCV RNA) measurements at days T1 and T4. Misclassified patients are x-marked.

LDA was used to classify patients on the basis of the viral HCV RNA at T1 (day one) and T4 (day four) (Fig 5A). It appears that early viral kinetics could be predictive of EVR/RVR since the resubstitution error is 21% (3 out of 14 patients). LDA was also used to analyze whether the early viral kinetics could be predictive of SVR using the 14 patients with available daily HCV RNA serum samples (Fig 5B). In this case, since the resubstitution error is 43% (6 out of 14 patients), it cannot be concluded that the HCV RNA at days T1 and T4 are able to predict SVR. Therefore, there is no association between SVR and viral kinetics earlier than week 1 after initiating therapy.

### Association between reduced serum HCV RNA levels after one month from starting therapy and SVR

**Fig 6:**
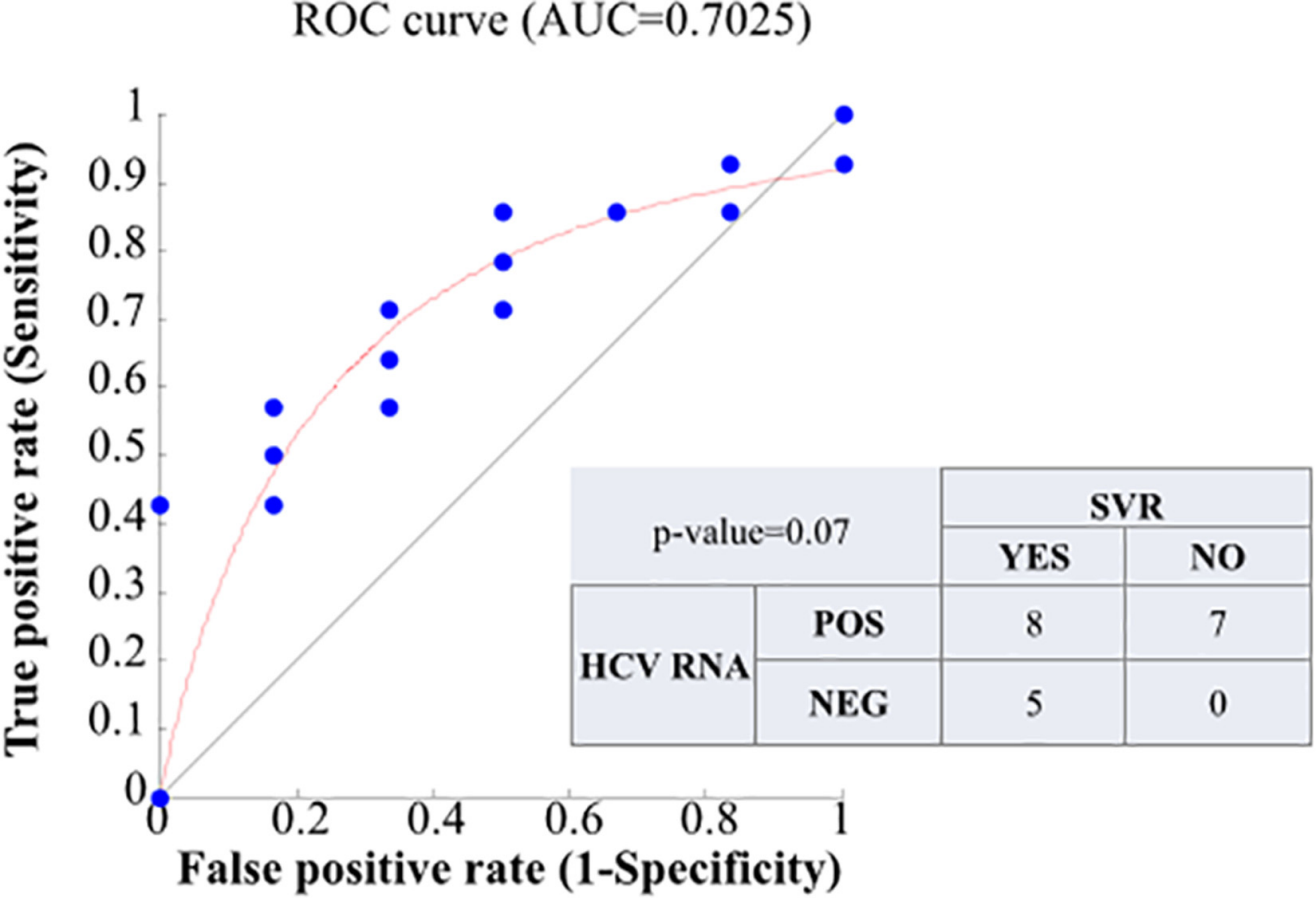
Receiver-operating characteristics ROC curve on the 20 patients’ HCV RNA measurements at month 1 after starting therapy. AUC = area under the ROC curve = 0.7. The contingency table for the two-tailed Fisher’s exact test is reported: the p-value = 0.07 indicates that the SVR response is quite statistically and significantly different in patients with detectable HCV RNA at the first month compared to patients with undetectable HCV RNA.

Evaluating the association between HCV RNA levels at T1m, T2m, and T3m after starting therapy and SVR, ROC analysis, which was calculated including only the HCV RNA values obtained by ART, gave no statistically significant results (data not shown). Instead, when we included the HCV RNA “not detected” value provided by the US method at T1m in the analysis, the area under the ROC curve was 0.70 (Fig 6), and the Max Efficiency cut-off value was calculated as 1.2 IU/ml.

In the inset of the same figure we show the Fisher’s exact test, to assess if an undetectable level of HCV RNA viraemia (with the US method) at T1m can be predictive of SVR (p-value = 0.07). Five patients with undetectable HCV RNA by the US method at T1m achieved SVR. Overall, PPV was 100%, NPV 46.7%, sensitivity 38.4%, and specificity 100%. Six out 8 patients with undetectable HCV RNA measured with ART at T1m achieved SVR.

## Discussion

Response-guided therapy (RGT), which was introduced to minimize exposure to new DAA drugs and optimize response is still widely used [28] and not only for HCV 1 genotype [29].

In the cases of TPV and BCV, only a small group of patients (naïve, non-cirrhotic, and those who reached RVR and eRVR,) could benefit from RGT and be eligible for a shortened duration of therapy. Furthemore, some studies, which have been carried out to assess whether SVR12w and SVR24w, have comparable results on predicting SVR in HCV infected patients treated by HCV NS3/4A protease inhibitors with PEG-IFN/RBV gave discordant results [30, 31].In fact Chen [30] observed an excellent concordance between SVR12w and SVR24w in predicting the success of the therapy, whereas Kanda [31] observed that it is better to use SVR24w for predicting SVR, especially in patients treated with simeprevir based-regimes. A greater-sensitivity HCV RNA assay could help to overcome this discrepancy as suggested by Kanda.

In this retrospective study we analyzed different virological parameters, to establish their ability to predict SVR achievement in TPV/PEG-IFN/RBV treatment.

The baseline HCV RNA level, which appears to be a good predictor in SOC therapy to achieve an RVR and that allowed for a shorter treatment of 24 weeks [32] did not have the same predictive value in our group of patients. Furthermore, statistical analysis demonstrated a correlation between HCV RNA values at early phases of treatment with EVR/RVR but no correlation with SVR (Fig 5 and S1 Fig).

Viral kinetic analysis based on a mathematical and statistical model of PCA demonstrated that the slope values could predict RVR, but not SVR. A considerable percentage (57.1%, 4/7) of patients achieved SVR, even without RVR: 21.4% of the RVR patients did not reach SVR.

IL28B rs12979860 genotype did not predict SVR response, most probably because most of the patients (91.3%; n = 21/23) had a SOC therapy treatment failure and so they had been preselected as C/T or T/T genotypes.

A promising parameter in SVR prediction was the undetectable residual viral load detected by the US method after T1m of treatment. The ROC analysis showed that results of HCV RNA “not detected” by the US method at T1m of treatment were strongly associated with SVR (0.71 under curve area), whereas no association was found when we considered the values at T12w after starting therapy (data no shown).

The results of the US method on low residual viraemia samples may have important clinical implications for monitoring viral response and the outcomes of response-guided therapy. In particular, 33.3% (3/9) of the patients with a minimal residual viral load (HCV RNA detected) by ART and by the US method at T4w did not achieve SVR. This suggests that the use of less sensitive qualitative assays to monitor virological response might lead to inappropriate discontinuation of treatment at week 24 with the risk of compromising SVR achievement. However, being positive at US HCV RNA at T1m cannot be used as a stopping rule, because of its low NPV (46.7%). Instead the PPV of undetectable US HCV RNA is high (100%), opening the possibility of considering shortened treatment duration.

Our data are in accordance with those described by Harrington et al. [17], who pointed out the clinical relevance of noting that those who had a detectable, but not quantifiable HCV RNA (HCV RNA <25 IU/ml, but >9.3 IU/ml) had a reduced SVR rate compared to those with undetectable HCV RNA at the same point.

Our findings corroborate the results obtained by Kinai, who demonstrated the correlation between undetectable RNA “calculated at T4w by US and SVR in SOC therapy [33]. The relevance of method sensitivity in monitoring the therapy represents an open question regarding new [34], and second generation DAA [35]. Our data apparently differ from that of Vermeheren et al. [14]. These authors analyzed the importance of HCV RNA detected <12 for the prediction of SVR, and they concluded that these values < LLOQ were not important for adopting a shortened therapy when they were introduced in RVR/EVR assessment, since most of the patients with “detected <12 IU/ml” at T4w who had a shortened therapeutic regimen achieved SVR. However, in this study **no** calculation of residual viral load was made, so we do not know if these values “detected <12 IU/ ml” corresponded to real HCV RNA positivity. Furthermore, since the authors did not provide any information about whether patients were naive or had failed previous antiviral therapy, other factors that could influence the success of treatment could not be excluded. Furthermore, our findings on the predictive power of the US HCV RNA value corroborate the results described by Nishida [12] and Ogawa [35] concerning antiviral triple therapy with simeprevir, peg-interferon and ribavirin.

Our study has several limitations. One is that the US method was not tested in duplicate or triplicate to address the potential impact of assay imprecision. Repeated testing was not possible due to limited sample volume. This would be necessary to validate a method, even though High specificity and confirmation of positive results on samples with a low IU input viral load were observed when several replicates were made with WHO standard. Although the number of samples analyzed with the US method was too small to draw definitive conclusions, the relevance of the US method viral load should be taken into account when identifying a predictive marker for SVR, and to enable better tailoring of the duration of treatment [36] even in new interferon-based DAA therapy regimens.

Furthermore the use of triple therapy with peg-IFN-alpha, RBV and TPV is not up to date, since newer generation NS3 PIs (simeprevir, paritaprevir/ritonavir), and other DAA targeting other HCV proteins, like NS5A (daclatasvir, ledipasvir, ombitasvir), and NS5B polymerase (sofosbuvir, dasabuvir), have entered the anti-HCV scenario, making first-generation PIs of secondary importance. However, this study may be considered proof of concept. The results obtained with US protocol could be employed as a model in other therapeutic regimens based on newer DAA, eventually making a decision to shorten the treatment possible.

Another limitation is the small number of patients included in the study; this was mainly due to the fact that telaprevir in Italy has been in use for a short time, and patient access to this DAA was limited by very restrictive clinical indications, so the number of patients eligible for the study was limited.

In conclusion, US HCV RNA parameter could be included in a algorithm to further individualize treatment strategies and minimize risk of adverse effects observed in new interferon-based DAA therapies and to reduce the cost of therapy without losing its efficacy.

## Supporting Information

S1 Data Efficacy of antiviral treatment definition. (DOCX)

S2 Data HCV RNA ultrasensitive method description. (DOCX)

S1 Fig Boxplot of HCV RNA baseline measurements (Log values) for SVR and no-SVR patients. The resulting p-values of a two-sided Wilcoxon rank sum test performed between the SVR and no SVR patients for HCV RNA baseline measurements are indicated.
(TIF)

S2 Fig Linearity of HCV RNA calibration of the modified calibration curve. The modified calibration curve is composed of 5 calibrators adjusted to 1,000, 200, 40, 8, and 4 IU/ml (3, 2.3, 1.6, 0.6 Log IU/ml, respectively), analyzed in 3 replicates for each point, in each run. **B**: WHO standard was diluted to 520, 104, 20.80, 4.16 IU/ml (2.71, 2.01, 1.31, and 0.61 Log IU/ml, respectively). The correlation between expected and observed results obtained with modified protocol (r^2^ = 0.91235) and with standard protocol (r^2^ = 0.9235, data no shown). The calculation of samples below 12 IU/ml measured with ART procedure was performed by extrapolating data towards low values of the standard curve. **C**: Performance characteristics of “the US method “observed with HCV RNA WHO standard, from 104 IU/ml to 0.20 IU/ ml (2.01, 1.31, 0.61, -0.08, -0.69 Log IU/ml, respectively). A total of 8 replicates, 3 times concentrated, were prepared and analyzed in 2 runs. Viral loads were correlated with expected values.
(TIF)
