# Supplementary material for: Ultrasensitive HCV RNA Quantification in Antiviral Triple Therapy: New Insight on Viral Clearance Dynamics and Treatment Outcome Predictors
Source: PLoS One. 2016 Aug 25;11(8):e0158989. doi: 10.1371/journal.pone.0158989 (PMC4999094; doi:10.1371/journal.pone.0158989)
Supplement: S1 Data — (DOCX) [file pone.0158989.s001.docx]

**S1 data-Efficacy of treatment**

Treatment response was categorized as follows: rapid virological response (RVR), an HCV RNA value <12 IU/ml at week 4; complete early virological response (cEVR), defined as HCV RNA<12 IU/ml after 4 and 12 weeks of treatment, and early virological response (EVR), when HCV RNA was >12 IU/ml at week 4 but <12 IU/ml at the week 12, and SVR, HCV RNA <12 IU/ml at 24 weeks after the end of treatment. The patients whose HCV RNA never became undetectable were classified as non-responders. “EOT” response was defined as HCV RNA <12IU/ml at the end of treatment.

Secondary efficacy assessments included virological breakthrough, defined as confirmed HCV RNA ≥12IU/ml after achieving HCV RNA< 12 IU/ml on treatment or when the viral level increased by 2 Log/IU/ml from nadir or to a level of more than 3 Log IU/ml after reaching an undetectable level during treatment. Relapsers were defined as confirmed HCV RNA ≥12 IU/ml during the 24 weeks after achieving HCV RNA <12 IU/ml at the end of treatment (EOT). Partial responders were subjects whose HCV RNA levels dropped by at least 2 Log IU/ml during treatment, but were still detectable.

All treatment was discontinued if patients had > 2 Log in HCV RNA 12 weeks after starting TPV or detectable (≥12 IU/ml) HCV RNA at weeks 24 or 36. Patients could discontinue therapy because of severe adverse effects.
