# Supplementary material for: Ultrasensitive HCV RNA Quantification in Antiviral Triple Therapy: New Insight on Viral Clearance Dynamics and Treatment Outcome Predictors
Source: PLoS One. 2016 Aug 25;11(8):e0158989. doi: 10.1371/journal.pone.0158989 (PMC4999094; doi:10.1371/journal.pone.0158989)
Supplement: S2 Data — (DOCX) [file pone.0158989.s002.docx]

**S2data -HCV RNA ultrasensitive method**

1.**Calibration curve** - The standard calibration curve spans from 800 to 800,000 IU/ml. The new calibration curve consisted of 5concentration levels with HCV RNA concentrations at: 1000, 200, 40, 8, and 4 IU/ml**.** Three replicates of each calibrator were included in each analytical session, together with negative and positive control. Sample extraction was performed with the Abbott *m2000rt*. Sample amplification and detection were conducted using the “open mode” software (Laboratory Defined Application). For validation, 10 consecutive runs were performed, considering the following parameters: slope, intercept and r^2^ [20]. The slope oscillated between -3.609 and- 3.504 (mean value: -3.556). The linearity assay based on the new calibration curve was tested with serial dilutions of the high positive control and the HCV standard (S2Fig A-B). The results indicated that the linearity was maintained up to 3,000 IU/ml (data not shown) with the US method. The WHO HCV RNA standard was detected in 100% of samples diluted down to 4 IU/ml, and a good correlation was observed with the obtained and expected values by using the US method (S2 Fig C).

2.**Increased input volume sample via ultracentrifugation**- To further increase the sensitivity of the assay, 3x concentrated input samples were prepared by ultracentrifugation 2.4ml of plasma was centrifuged at 20,000 x g for 90 min at 4°C. The excess volume of plasma supernatant was decanted and the remaining 0.8 ml volume with the resuspended pellet was subject to extraction and amplification and detection. The modified Abbott RealTi*me* HCV assay protocol the extended calibration curve combined with Laboratory Defined Application software and 3x concentrated input samples are referred to as “**ultrasensitive**” (US) method .

3. **Internal control**- The internal control was taken through the entire extraction and amplification procedure, as indicated in the manufacturer’s instructions, with the difference that the volume was reduced from 20.8 to 4.16 μl/sample to prevent amplification PCR inhibition in sample with low viral load.

4.” O**pen mode” software**- Amplification and detection were performed with the Abbott m2000rt equipped with “open mode“ software (Laboratory Defined Application) provided by Abbott Molecular. Amplification parameters used in the US method were the same as the ones used in the standard protocol. The types of results observed with the US method were as follows: a) quantified HCV RNA above 4IU/ml; b) detected <4 IU/ml, and c) not detected.

Performance characteristics of the US method were analysed using multiple replicates of a dilution panel, obtained by the HCV RNA WHO standard reagent (NIBSC code:06/100/2009, [www.nibsc.ac.uk](http://www.nibsc.ac.uk)). We performed twofold dilutions using HCV negative human plasma (Basematrix; Boston Biomedica Inc, Wbridwater, MA) **(**from 520 to 0.83 IU/mL). Seven replicates were prepared for each dilution. Analytical sensitivity (i.e., concentration of HCV RNA detected with a probability of 95%) was determined by PROBIT regression model. “The US method” protocol was applied to a series of residual clinical samples from 4,8, and 12 weeks of treatment with TPV/PEG-IFN/RBV having ART HCV RNA level “<12IU/ml, HCV RNA detected” or “HCV RNA not detected” were tested on the US method . Plasma samples stored at -80°C were thawed only once. Agreement was assessed between ART procedure and the modified US method. Results were defined as concordant “positive” when HCV RNA was quantifiable or <12IU/ml detected by ART and quantifiable or <4 IU/ml by the US method, or when HCV RNA was undetectable by both assays. Results were defined as discordant when ART detected HCV RNA detected <12/ml, and the US method gave “HCV RNA not detected” results or *vice versa*. The analytical sensitivity (i.e. the concentration of HCV RNA detected with response probability of 95%) was calculated by PROBIT analysis, and indicated a limit of detection sensitivity (LOD) of the US method of 1.2 IU/ml. Linearity, precision and accuracy were also determined. The correlation was assessed by testing WHO standard with ART and the US method and calculating the Pearson correlation coefficient (S2Fig.). For specificity, 20 HCV antibody and RNA samples were tested with the US method.
