# Supplementary figures and images for: Ultrasensitive HCV RNA Quantification in Antiviral Triple Therapy: New Insight on Viral Clearance Dynamics and Treatment Outcome Predictors
Source: PLoS One. 2016 Aug 25;11(8):e0158989. doi: 10.1371/journal.pone.0158989 (PMC4999094; doi:10.1371/journal.pone.0158989)

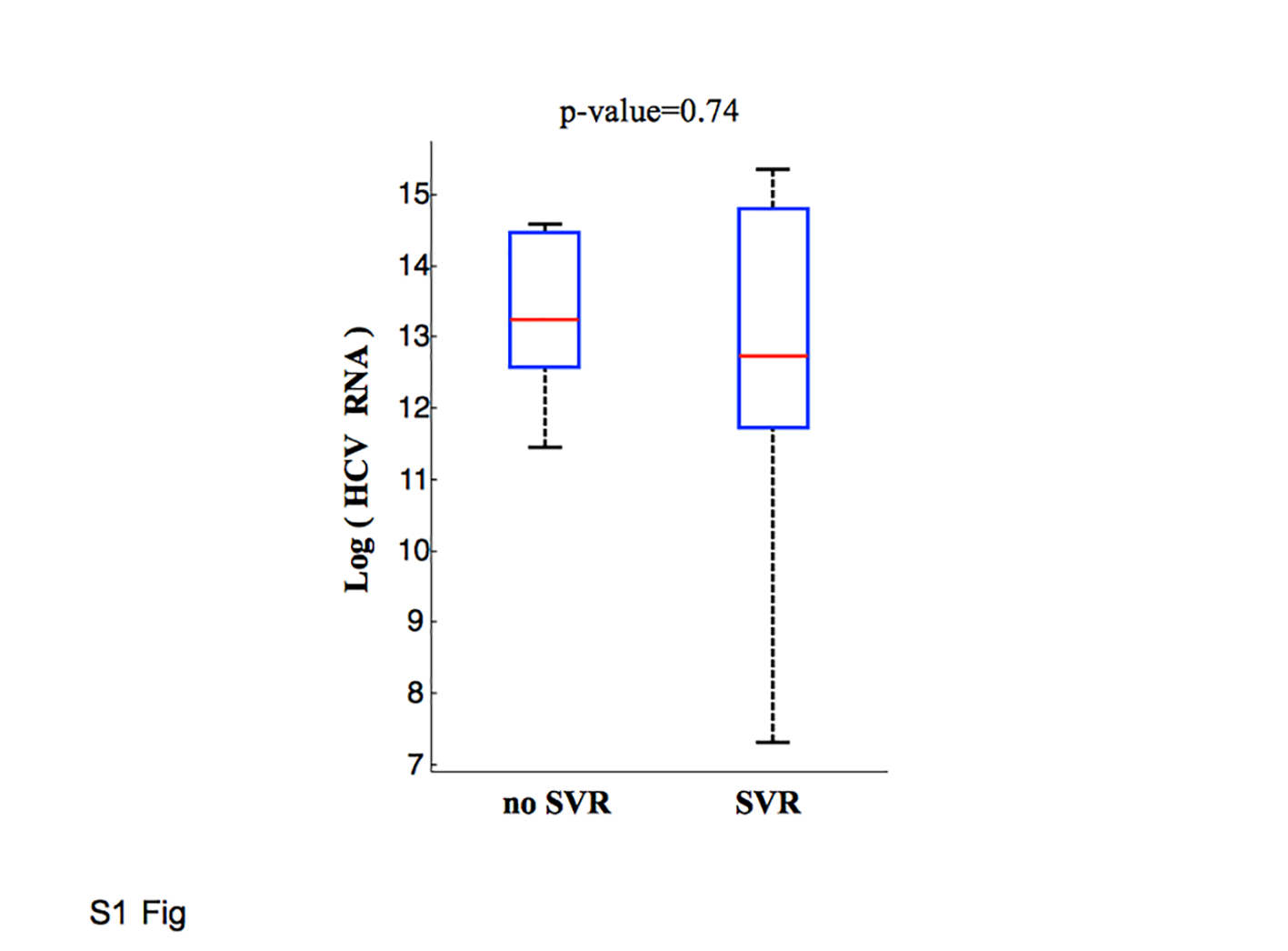

Supplement: S1 Fig — The resulting p-values of a two-sided Wilcoxon rank sum test performed between the SVR and no SVR patients for HCV RNA baseline measurements are indicated. (TIF) [file pone.0158989.s003.tif]

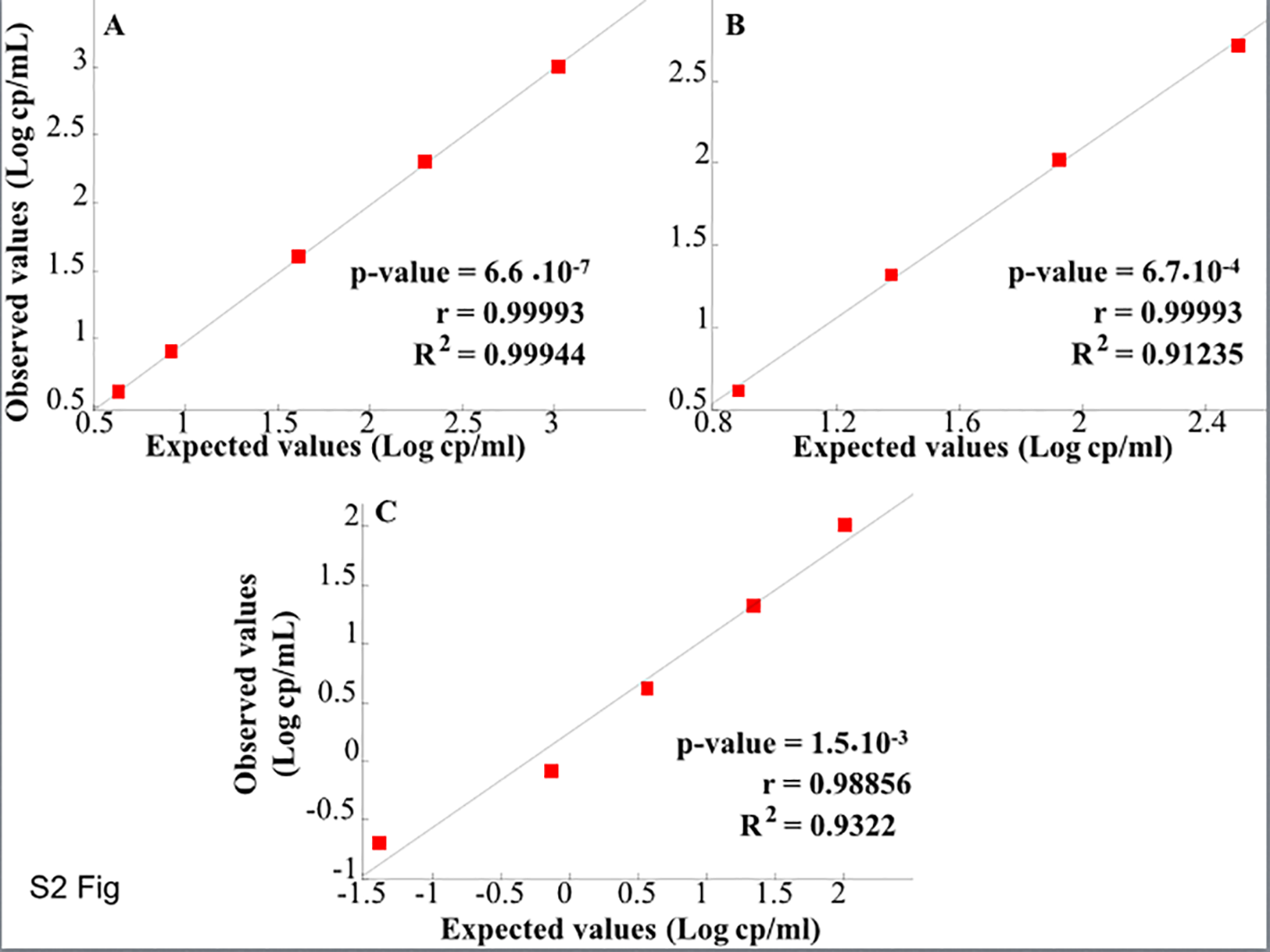

Supplement: S2 Fig — The modified calibration curve is composed of 5 calibrators adjusted to 1,000, 200, 40, 8, and 4 IU/ml (3, 2.3, 1.6, 0.6 Log IU/ml, respectively), analyzed in 3 replicates for each point, in each run. B: WHO standard was diluted to 520, 104, 20.80, 4.16 IU/ml (2.71, 2.01, 1.31, and 0.61 Log IU/ml, respectively). The correlation between expected and observed results obtained with modified protocol (r2 = 0.91235) and with standard protocol (r2 = 0.9235, data no shown). The calculation of samples below 12 IU/ml measured with ART procedure was performed by extrapolating data towards low values of the standard curve. C: Performance characteristics of “the US method “observed with HCV RNA WHO standard, from 104 IU/ml to 0.20 IU/ ml (2.01, 1.31, 0.61, -0.08, -0.69 Log IU/ml, respectively). A total of 8 replicates, 3 times concentrated, were prepared and analyzed in 2 runs. Viral loads were correlated with expected values. (TIF) [file pone.0158989.s004.tif]
